# Ag surface segregation in nanoporous Au catalysts during CO oxidation

**DOI:** 10.1038/s41598-018-33631-4

**Published:** 2018-10-12

**Authors:** Giorgio Pia, Elisa Sogne, Andrea Falqui, Francesco Delogu

**Affiliations:** 1 Dipartimento di Ingegneria Meccanica, Chimica, e dei Materiali, Università degli Studi di Cagliari, via Marengo 2, 09123 Cagliari, Italy; 2 King Abdullah University of Science and Technology (KAUST), Biological and Environmental Sciences and Engineering (BESE) Division, NABLA Lab, 23955-6900 Thuwal, Saudi Arabia

## Abstract

The present study focuses on the modification of surface compositional profiles induced in nanoporous (NP) Au catalysts by the catalytic oxidation of carbon monoxide to carbon dioxide in the presence of oxygen. The phenomenon has deep implications concerning the catalytic behavior of NP Au foams in particular, and more in general for the design of more efficient catalysts. Aimed at gaining deeper insight into the mechanisms governing surface segregation, we exposed NP Au foams containing residual Ag to a mixture of gaseous carbon monoxide and oxygen at different temperature. Structural and surface composition analyses pointed out the concomitant occurrence of both NP Au coarsening and Ag surface segregation processes. Experimental findings suggest for Ag surface segregation a two-stage kinetics. During the initial, rapid coarsening of the NP Au structure, Ag surface segregation is mediated by surface rearrangements, which allow the Ag atoms to reach the surface at anomalously fast rate. As coarsening decelerates, the slower diffusion of buried Ag atoms towards the surface predominates, due to favorable chemical interactions with adsorbed oxygen. This novel mechanism’s understanding can benefit strategic areas of science and technology.

## Introduction

Last ten years have seen a flowering of interest in the study of monolithic nanoporous (NP) Au foams^1–3^. These materials display their most distinctive feature from a structural point of view. Their structure comprises two bi-continuous complementary networks of pores and ligaments interconnected by relatively massive nodes, which combine their irregular morphologies into a disordered three-dimensional maze of open cells^1–3^. Whereas inter-percolation of void and matter results in high surface area-to-volume ratio, characteristic lengths on the nanometer scale result for ligaments and pores in small curvature radii^4^. In turn, these features bring to stepped surfaces with unusually high concentration of under-coordinated atoms at kink and edge sites^4^.

Large specific surface area and high degrees of coordination unsaturation for surface atoms deeply affect the whole spectrum of surface processes, imparting NP Au foams a set of unique physical and chemical properties^4–6^. For instance, the capability of supporting simultaneously propagating and localized modes of surface plasmon resonance renders NP Au the ideal substrate for surface-enhanced Raman scattering, enabling the probe and identification of low concentration molecules in chemical and biological systems^7–9^. Similarly, tuning the solid-liquid interfacial tension by suitable external electric potential allows the active control of imbibition processes, transforming NP Au foams into electrocapillary pumps^10^. In the same manner, the reversible modification of surface stress induced by surface-adsorbate interactions allows converting chemical energy into a mechanical action, making NP Au work as a surface-chemistry-driven actuator^11,12^. Lastly, NP Au foams behave unexpectedly as excellent catalysts for oxidation processes^4,13–15^.

Concerning catalysis, experimental evidence strongly suggests that large specific surface area and stepped surfaces are not the only influential factors. Indeed, catalytic performances are also affected by the surface concentration of residual heteroatoms surviving the chemical and electrochemical de-alloying processes used to fabricate NP Au foams^16–19^. In this respect, experimental findings reveal that the catalytic transformation of carbon monoxide (CO) into carbon dioxide (CO_2_) induces an increase of Ag and Cu surface concentration in NP Au foams obtained, respectively, from Ag-Au and Cu-Au parent alloys^20^. Moreover, Ag surface segregation enhances the catalytic activity, indicating a pivotal role of the residual Ag in the activation of molecular oxygen (O_2_) beneficial to the catalytic CO oxidation^20^. The evidence that catalytic transformations promote the coarsening of NP Au foams^6,20–25^ and that chemically active surface sites also participate in surface diffusion processes mediating coarsening^6,20–25^, further complicates the conceptual framework.

Under these circumstances, characterizing the kinetics of Ag surface segregation in NP Au foams exposed to reactive CO-O_2_ gaseous mixtures represents a crucial step to advance fundamental knowledge and enable the design of superior NP Au catalysts. Focusing exactly on this issue, we provide sound experimental evidence of the deep link between the Ag surface segregation and coarsening processes that occur simultaneously in NP Au foams throughout the catalytic CO oxidation.

With this aim, we performed catalytic experiments on NP Au foams fabricated by chemical dealloying from a parent Ag_70_Au_30_ alloy. Specifically, we exposed the NP Au foams to a reactant gas mixture consisting of 1% CO, 10% O_2_, and 89% nitrogen (N_2_) under flow conditions at temperatures between 273 and 303 K (see Supporting Information SI. 1 for details).

## Results and Discussion

**Figure 1:**
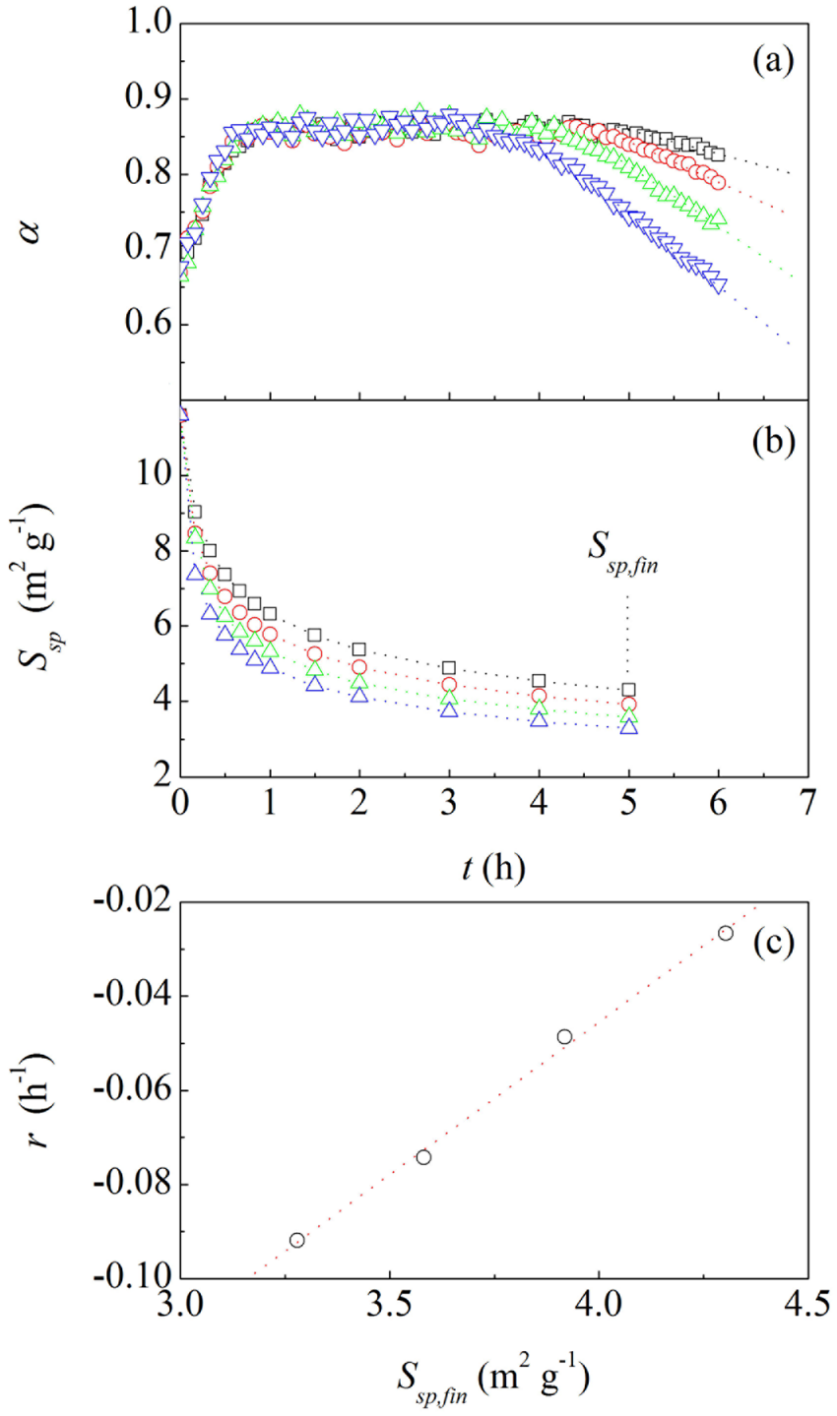
Relationship between catalytic transformation and coarsening. (**a**) The conversion degree of CO to CO_2_, *α*, as a function of time *t*. (**b**) The specific surface area, *S*_*sp*_, of NP Au catalysts as a function of time, *t*. Data refer to NP Au foams used in catalytic runs performed at 273 (□), 283 (), 293 () and 303 () K. The vertical dotted line marks the *S*_*sp*_ values, *S*_*sp*_,_*fin*_ attained after 5 h of exposition to reactive gases. (**c**) The rate of catalyst activity reduction, *r*, as a function of the final specific surface area, *S*_*sp*_,_*fin*_. Best-fitted line is also shown.

The exposure of NP Au catalysts to gaseous reactants caused the efficient oxidation of CO to CO_2_. Data shown in Fig. 1a indicate that catalytic performances are almost insensitive to temperature. A short induction period precedes the attainment of a steady state, characterized by an approximately constant conversion degree, *α*, of about 0.86. Accordingly, about the 86% of CO is converted into CO_2_ within the residence time of the reactant mixture in the reactor. In agreement with literature^6^, we observed that the catalyst activity starts declining after about 4 h, with an approximately linear *α* reduction from the steady-state value to smaller ones. The rate at which the catalytic activity declines, *r*, increases with temperature.

The degeneration of catalytic performances can be ascribed to structural modifications of NP Au foams caused by catalytic runs^6^, which are readily pointed out by the measured values of specific surface area, *S*_*sp*_, shown in Fig. 1b. In all cases, *S*_*sp*_ undergoes a monotonic decrease. The value attained after 5 h, hereafter considered as the final value, *S*_*sp*,*fin*_, decreases with temperature. As shown in Fig. 1a, after about 4 h the catalytic activity starts decreasing following a linear trend. The slope of the linear trend, *r*, provides a measure of the rate at which the catalytic activity decreases. The correlation between *r* and *S*_*sp*,*fin*_ emerging from Fig. 1c definitely connects the specific surface area with the catalyst efficiency.

**Figure 2:**
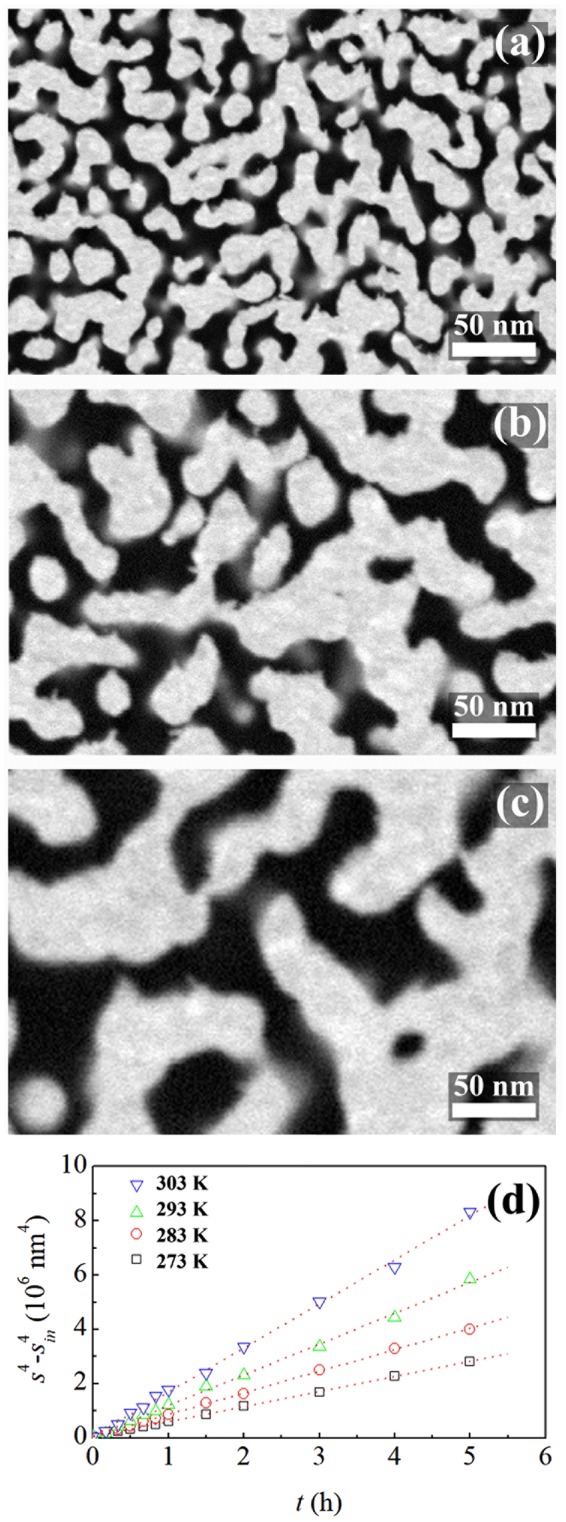
Kinetics of coarsening. SEM micrographs of NP Au foams exposed to reactive gases for (**a**) 0, (**b**) 2 and (**c**) 4 h at 273 K. (**d**) The logarithm of ${s}^{4}-{s}_{in}^{4}$ as a function of time *t*. Data refer to NP Au foams used in catalytic runs performed at 273 (□), 283 (), 293 () and 303 () K. Best-fitted lines are shown.

The specific surface area decreases because of the coarsening processes involving ligaments and pores of NP Au catalysts. SEM micrographs in Fig. 2a–c clearly show that the average ligament diameter, *s*, of NP Au foams exposed to reactive gases at 273 K grows from the initial average value, *s*_*in*_, of 15 nm to about 28 and 38 nm after 2 and 4 h respectively. Data plotted in Fig. 2d reveal that the temperature affects the coarsening rate, but not its kinetic features. Indeed, the ligament diameter, *s*, grows invariably according to a fourth-power law, which is the expected dependence for coarsening processes mediated by diffusion of surface atoms^26–29^. Accordingly, the fourth power of *s*, *s*^4^, increases linearly with time.

**Figure 3:**
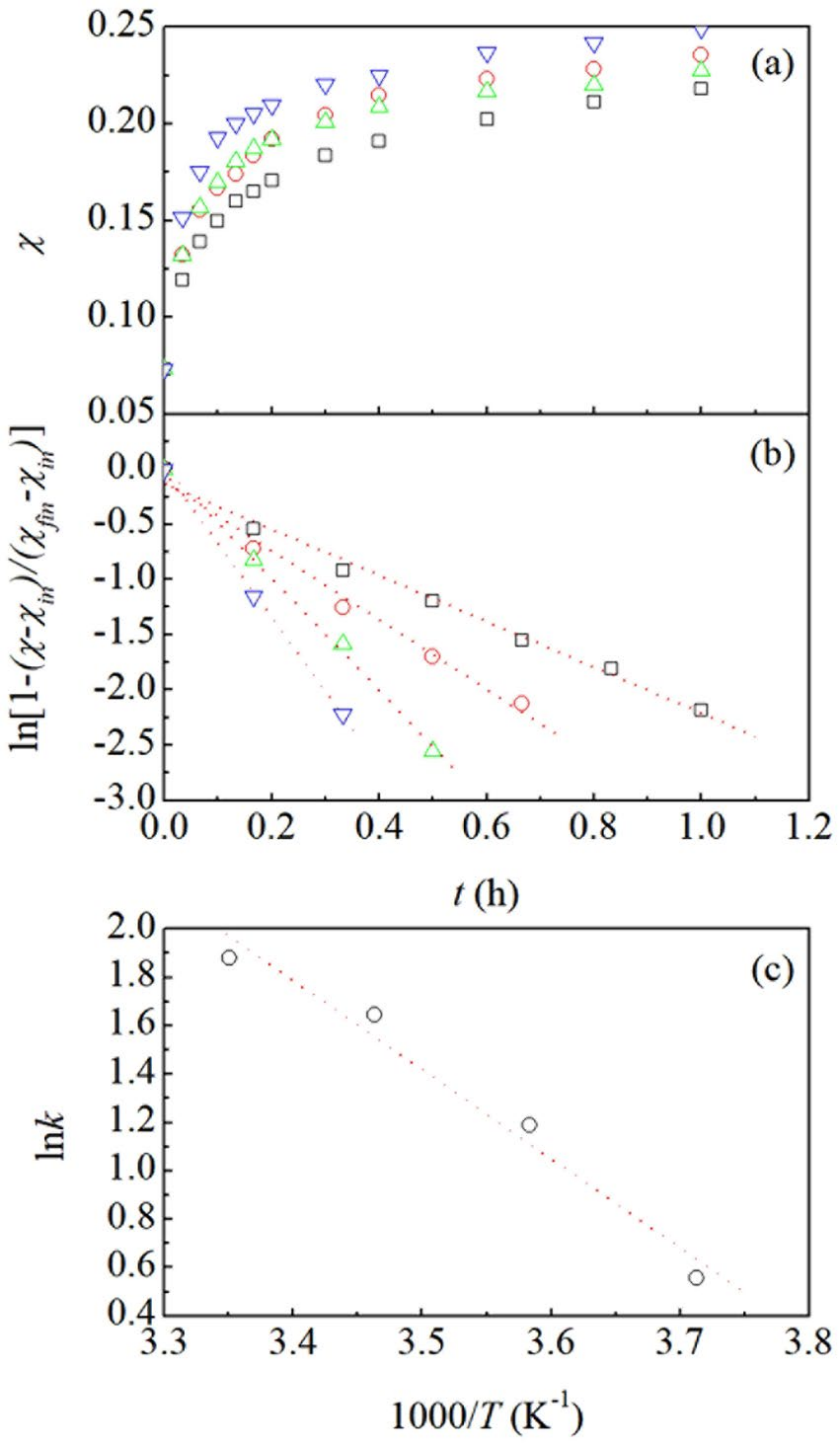
Kinetics of Ag segregation at NP Au surface. (**a**) The relative surface concentration of Ag atoms, *χ*, as a function of time, *t*. (**b**) The logarithm of $1-(\chi -{\chi }_{in})/({\chi }_{fin}-{\chi }_{in})$, $\mathrm{ln}[1-(\chi -{\chi }_{in})/({\chi }_{fin}-{\chi }_{in})]$ as a function of time, *t*. Data refer to NP Au foams used in catalytic runs performed at 273 (□), 283 (), 293 () and 303 () K. Best-fitted lines are shown. (**c**) The logarithm of apparent rate constants, In *k*, as a function of the inverse of temperature, *T*. Best-fitted line is shown.

XPS measurements indicate that significant Ag surface enrichment accompanies coarsening (see Supporting Information SI. 2 for details). The Ag surface concentration was estimated on a relative basis by comparing the XPS spectra of different samples. Specifically, the relative surface concentration of Ag atoms was evaluated using the measured intensities of Ag(3d) and Au(4 f) peaks using tabulated sensitivity factors. Thus, information was obtained on the chemical composition of a surface layer about 3 to 4 nm thick. The relative Ag surface concentration was calculated as the ratio between the number of Ag atoms located in the surface layer and the total number of atoms in the surface layer. It can be seen from Fig. 3a that the relative surface concentration of Ag atoms, *χ*, increases monotonically with time, *t*. The rate of Ag surface segregation also increases with temperature, and the same happens for the final *χ* values, *χ*_*fin*_, which are in the range between 0.21 and 0.25. Since the total residual Ag concentration in pristine NP Au foams is equal to about 3.8 at. % (see Supporting Information SI. 2 for details), segregation processes involve, at most, only the 30% of residual Ag atoms. Nevertheless, relative Ag surface concentration undergoes a more than threefold increase.

No simple mathematical curve exhibits the ability of interpolating to a satisfactory extent the entire set of experimental points obtained at any given temperature. However, limitedly to *χ* values smaller than 0.18, we note that a suitable choice of parameters *χ*_*in*_ and *χ*_*fin*_ results in the linear plots of $\mathrm{ln}[1-(\chi -{\chi }_{in})/({\chi }_{fin}-{\chi }_{in})]$ as a function of time, *t*, shown in Fig. 3b. Whereas *χ*_*in*_ can be set equal to the initial value of about 0.073 in all cases, best *χ*_*fin*_ choices range approximately between 0.16 and 0.18.

Linear plots in Fig. 3b suggest for the first stages of Ag surface segregation induced by catalytic CO oxidation the kinetic equation

1 $$\chi ={\chi }_{in}+({\chi }_{fin}-{\chi }_{in})\,[1-\exp (-k\,t)],$$

where *χ*_*in*_ is the initial relative Ag surface concentration, *χ*_*fin*_ the relative Ag surface concentration attainable at the end of the first stage, *i*.*e*. when *χ* = 0.18, and *k* is the apparent rate constant. Indeed, Eq. 1 can be readily rearranged into

2 $$\mathrm{ln}(1-\frac{\chi -{\chi }_{in}}{{\chi }_{fin}-{\chi }_{in}})=\,-\,k\,t,$$

which satisfactorily describes the observed linearity in Fig. 3b.

Linearity is particularly convincing for data sets coming from experiments performed at 273 and 283 K, which comprise 7 and 5 points respectively. The number of points reduces to 4 and 3 for catalytic runs carried out, respectively, at 293 and 303 K. In these cases, the sampling time of 10 min utilized for investigating the kinetics during the first hour of experiment is no longer satisfactory because of the rate enhancement of Ag surface segregation with temperature. Unfortunately, reducing the sampling time to 5 min or less was not viable because of the comparable length of analytical procedures.

Based on Eq. 2, the slope of the linear plots in Fig. 3b corresponds to the apparent rate constant, *k*, of the Ag surface segregation process. As shown in Fig. 3c, points arrange approximately linearly when the logarithm of the apparent rate constant, In *k*, is plotted as a function of the inverse of temperature, *T*^−1^. This suggests for *k* an Arrhenius-like dependence on *T*. It follows that the slope of the semi-logarithmic plot provides a measure of the ratio ${E}_{a,CO+{O}_{2}}/R$ between the apparent activation energy for Ag surface segregation processes in the presence of CO, ${E}_{a,CO+{O}_{2}}$, and the universal gas constant, *R*. The linear interpolation of data in Fig. 3c indicates for ${E}_{a,CO+{O}_{2}}$ the best-fitted value of about 30.6 kJ mol^−1^.

This value is definitely smaller than the one of about 104.5 kJ mol^−1^ relative to bulk diffusion of Ag atoms in Au^30^. It is also approximately half the value of about 62 kJ mol^−1^ associated with the surface diffusion of individual Au atoms^26^. In contrast, it is relatively close to low activation barriers typical of atomic diffusion along grain boundaries^31,32^.

The ${E}_{a,CO+{O}_{2}}$ value is also quite smaller than the apparent activation energy, ${E}_{a,{O}_{2}}$, of about 78.3 kJ mol^−1^ governing Ag surface segregation in NP Au foams exposed to a gaseous mixture of 10% O_2_ and 90% N_2_, which we also investigated for comparison purposes (see Supporting Information SI. 3 for details). Ag surface segregation in the absence of CO takes place on time scales approximately 10 times longer than in the presence of CO. Since no catalytic transformation can occur, and no modification of surface composition is observed in the presence of gaseous N_2_ only (see Supporting Information SI. 3 for details), Ag surface segregation in the absence of CO can be reasonably ascribed to interactions between the surface of NP Au foams and gaseous O_2_. In this respect, it is worth noting that the ${E}_{a,{O}_{2}}$ value is almost perfectly half way between the value of about 104.5 kJ mol^−1^ for bulk diffusion of Ag atoms in Au^30^ and the one of about 62 kJ mol^−1^ for Au surface diffusion^26^. This suggests that Ag surface segregation in the absence of CO and in the presence of O_2_ does not depend exclusively on the effects of the Ag concentration gradient between the interior and the surface of ligaments. Caused by the selective Ag dissolution processes that have involved preferentially the surface layers of NP Au foams, the gradient would result in the diffusion of Ag atoms from the interior of ligaments to their surface. In such case, the apparent activation energy ${E}_{a,{O}_{2}}$ would be quite close to the value of about 104.5 kJ mol^−1^ for bulk diffusion of Ag atoms in Au. The fact that ${E}_{a,{O}_{2}}$ is significantly lower indicates that the Ag surface segregation process is affected by the interactions between gaseous O_2_ and the surface of NP Au foams.

Atomic diffusion governed by concentration gradients, even if possibly biased by gaseous O_2_, cannot explain the high rate of the Ag surface segregation processes taking place in the presence of CO. Therefore, other factors are acting in such case, somehow facilitating the migration of Ag atoms from the bulk of ligaments to their surface.

In this regard, it is worth noting that the structure of NP Au foams exposed to gaseous phases in the absence of CO remains substantially unaltered (see Supporting Information SI. 3 for details). For instance, in pristine NP Au foams exposed to the 10% O_2_ and 90% N_2_ gaseous mixture at 303 K ligaments reach a diameter around 20 nm after 50 h. In experiments performed at the same temperature, but in the presence of gaseous N_2_ only, the ligament diameter remains at the initial value of about 15 nm. Thus, NP Au foams undergo coarsening and Ag surface segregation processes involving the same time scale only in the presence of CO.

The above-mentioned correlation between Ag surface segregation and coarsening in NP Au catalysts during CO oxidation suggests that the two processes are somehow coupled. In particular, it seems that the atomic-scale processes governing mass redistribution on ever-increasing length scales enhance Ag surface segregation, promoting the exposition to gaseous phase of Ag atoms initially buried in the bulk of ligaments and nodes. Following this hypothesis, it can be surmised that an increasing number of initially buried Ag atoms is brought to the surface of NP Au foams by Au surface diffusion phenomena. Subsequently, the Ag atoms that have segregated to the surface can undergo surface diffusion in the presence of reactive gases. It follows that the number of Ag atoms displaced from their initial buried position, and finally brought to the surface, can be expected to be proportional to the total mass, or the total volume, of NP Au affected by morphological evolution. Therefore, the total mass, or the total volume, of NP Au foam involved in structural modification can potentially correlate with the relative Ag surface concentration.

**Figure 4:**
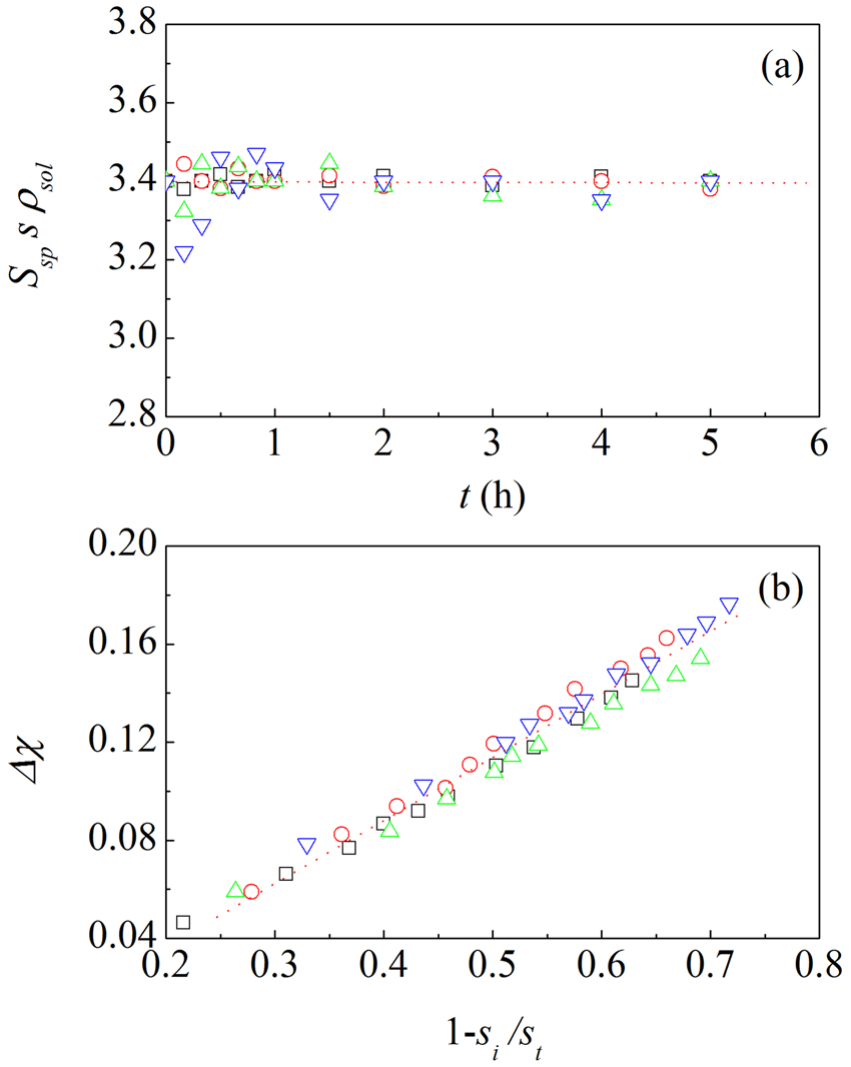
The scaling between the mass of NP Au affected by coarsening and Ag surface concentration. (**a**) The quantity *S*_*sp*_ *s ρ*_*sol*_ as a function of time, *t*. Best-fitted horizontal line is shown. (**b**) The increase of relative surface concentration of Ag atoms, Δ*χ*, as a function of the quantity $1-{s}_{in}/s$ at time *t*. Data refer to NP Au foams used in catalytic runs performed at 273 (□), 283 (), 293 () and 303 () K. Best-fitted lines are shown.

To a first approximation, the NP Au volume involved in coarsening can be estimated referring to the specific surface area, *S*_*sp*_ (see Supporting Information SI. 4 for details). In this respect, it is preliminarily worth noting that *S*_*sp*_ is deeply connected with the average thickness, *s*, of the ligaments constituting NP Au foams and the density of bulk Au, *ρ*_*sol*_. Indeed, as shown in Fig. 4a, the plot of the product *S*_*sp*_ *s ρ*_*sol*_ as a function of time defines the horizontal line *S*_*sp*_ *s ρ*_*sol*_ = *C*. The best-fitted *C* value is equal to 3.4 ± 0.2. According to literature^33,34^, such value indicates for the NP Au structure a definite gyroidal character. Despite coarsening, NP Au structures keep the gyroidal character approximately throughout the catalytic runs.

Upon these conditions, simple assumptions on the redistribution of mass during coarsening allow to derive for the volume, *v*_*c*_, of NP Au foams affected by shape evolution the expression (see Supporting Information SI. 4 for details):

3 $${v}_{c}\approx \frac{{m}_{tot}}{{\rho }_{sol}}(1-\frac{{s}_{in}}{s}),$$

where *s*_*in*_ and *s* represent the ligament thickness in pristine NP Au foams and after any given time *t* respectively, and *m*_*tot*_ is the total mass of Au. The increase of Ag surface atoms, Δ*χ*, calculated by the difference *χ* − *χ*_*in*_ between the relative concentration of Ag atoms at the surface of NP Au foams after any given time *t*, *χ*, and in pristine material, *χ*_*in*_, is shown in Fig. 4b as a function of 1 − s_*in*_/*s*. The plot is approximately linear, thus revealing a proportionality between the degree of Ag surface enrichment and the volume involved in coarsening. Therefore, relative Ag surface concentration scales with the mass of Au displaced during coarsening.

**Figure 5:**
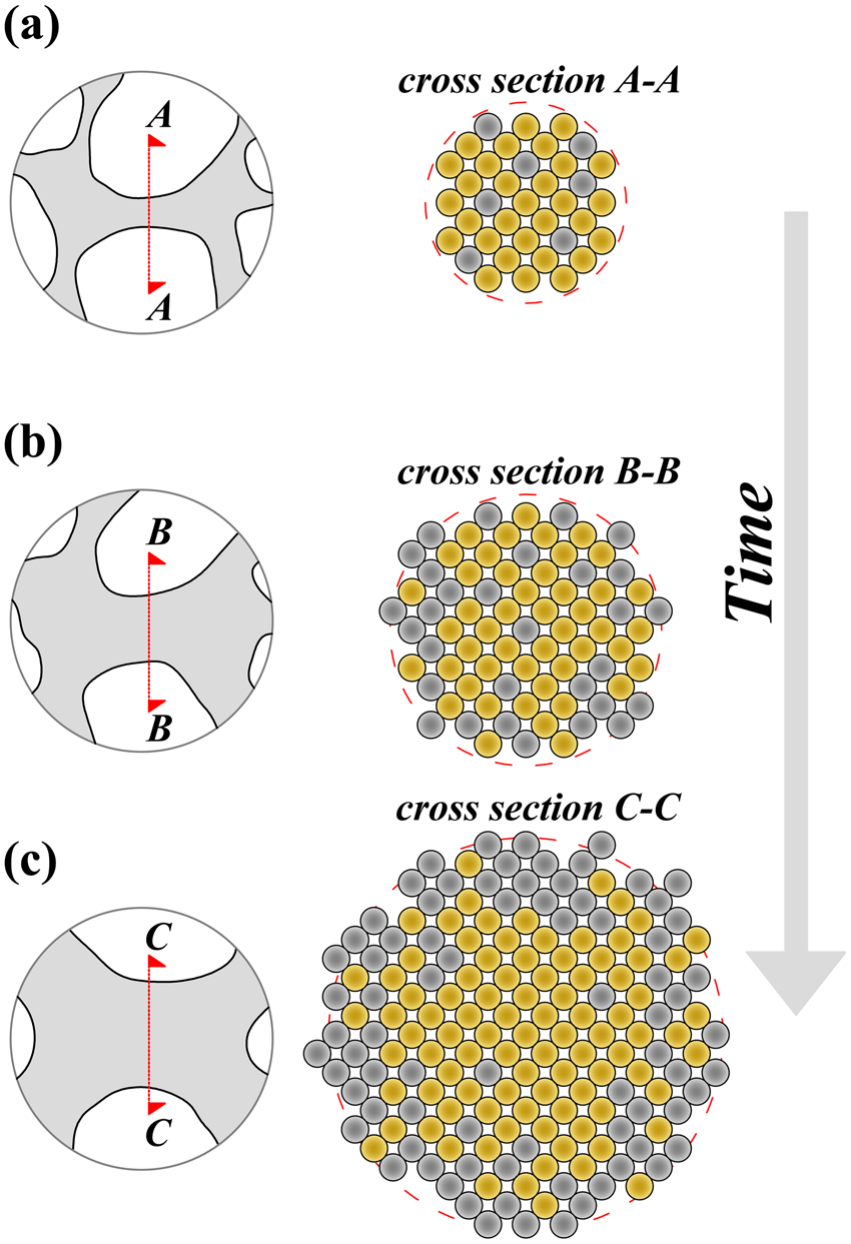
Schematic description of coarsening-mediated Ag surface segregation processes. The ligament diameter increases with time due to the displacement of Ag () and Au () atoms. The surface of the coarsening ligament progressively enriches in Ag atoms.

This latter evidence demonstrates that Ag surface segregation and coarsening processes taking place in the presence of CO are intimately coupled. Under these circumstances, it seems reasonable to hypothesize that Ag surface segregation in NP Au foams exposed to gaseous mixtures of 1% CO, 10% O_2_, and 89% N_2_ occurs *via* simultaneous, cooperative displacements of Ag and Au atoms. The surface mobility of Au atoms during the catalytic CO oxidation underlies the whole mechanism allowing gradual Ag surface enrichment. The latter can be described according to the consecutive stages schematically depicted in Fig. 5. In agreement with literature^6^, surface diffusion preferentially involves atoms located at chemically active surface steps on the topmost surface layer. As atomic displacements occur, smaller ligaments undergo a progressive layer-by-layer thinning accompanied by the corresponding coarsening of pores^6^. During the gradual refinement of ligaments, initially buried Ag atoms are exposed to the gaseous phase. Due to the favorable chemical interactions of Ag atoms with O_2_, and the corresponding lowering of the Gibbs free energy, Ag atoms remain at the surface, showing no tendency to occupy buried positions. Thus, coarsening is associated with the irreversible Ag surface enrichment of the NP Au catalyst.

The rate difference between coarsening-mediated and O_2_-induced Ag surface enrichment can satisfactorily explain the two-stage kinetics observed during the catalytic CO oxidation. Coarsening controls the first stage. Correspondingly, cooperative rearrangements determine the rapid decrease of specific surface area and the simultaneous increase of relative Ag surface concentration. Conversely, the second stage involves the diffusion of individual Ag atoms from the bulk to the surface biased by the chemical interactions between Ag and gaseous O_2_. Although present also during the first stage, this latter process is significantly slower than the coarsening-induced surface enrichment in Ag. Thus, it becomes predominant only in the second stage.

## Conclusions

In summary, we exposed NP Au catalysts to reactant gas mixtures consisting of 1% CO, 10% O_2_, and 89% N_2_ under flow conditions. Structural and chemical analyses reveal that NP Au foams undergo significant Ag surface segregation. Ag surface enrichment occurs in two stages. A rapid increase of relative Ag surface concentration takes place initially, followed by a much slower increment. Experimental findings suggest that the initial stage is connected with coarsening, whereas the second stage can be ascribed to adsorbate-surface interactions. A relatively simple kinetic scenario emerges from experiments. Catalytic transformations proceed preferentially at coordinatively unsaturated surface sites, thus enhancing the mobility of surface species. Consequent mass transport processes driven by the thermodynamic tendency of smoothing high-curvature surfaces and reduce total surface area determine remarkable shape evolution, affecting a significant volume fraction of NP Au catalysts. In particular, coarsening involves the progressive thinning, and the final pinch-off, of some ligaments and the redistribution of atoms on the free surface. Consequently, an increasing number of initially buried Ag atoms is exposed to the gaseous phase. This occurs at rates significantly higher than those allowed by the diffusion of Ag in bulk Au. Exposed Ag atoms establish favorable chemical interactions with O_2_. This allows them to remain at the surface for the whole duration of catalytic runs, showing no tendency to be buried again. Thus, Ag surface segregation initially proceeds in combination with coarsening. Once coarsening decelerates, Ag surface segregation becomes controlled by the diffusion of buried Ag atoms under the bias of O_2_-surface chemical interactions.

## Methods

We used small cylindrical pellets, about 1 mm thick and 1 cm in diameter, of a parent Ag_70_Au_30_ alloy prepared by mechanical alloying of elemental powders and subsequent annealing as starting material for fabricating NP Au foams. To this aim, we immersed the pellets in an aqueous solution of nitric acid at 70% to dissolve selectively Ag at room temperature. Chemical etching was followed by X-ray diffraction from pellets and argentometric titration of aqueous solutions. We interrupted the etching process after 6.5 h immersing the pellets in distilled water. Residual acid in pores was removed by water rinsing. Based on residual mass and pellet geometry, we calculated for our NP Au foams a relative density of about 0.294. The analysis of SEM and TEM images resulted in a similar estimate. We used SEM and TEM imaging analysis also for estimating the average ligament thickness in pristine NP Au foams and after exposition to reactive gases.

NP Au catalysts were exposed to reactants inside a tubular quartz reactor. We used a gas mixture of 1% CO, 10% O_2_, and 89% N_2_ flowing at a space velocity of 120 dm^3^ h^−1^ per catalyst mass unit. Pressure was kept constant at 1 atm. Catalytic tests were performed at 273, 283, 293 and 303 K. Relative amounts of CO and CO_2_ were measured online using a Shimadzu GC-8A gas chromatograph. Control experiments were performed under the same above-mentioned conditions using 10% O_2_ and 90% N_2_ gaseous mixtures and pure N_2_.

Relative surface composition was estimated by X-ray photoelectron spectroscopy (XPS) using a Perkin Elmer Phi 5600 ESCA spectrometer equipped with a Mg K_*α*_ X-ray source for excitation. We restricted the analysis to the binding energies of Ag(3d) and Au(4 f) electronic states, calibrated with respect to the C(1 s) peak at 284.6 eV. We calculated relative surface concentrations from measured intensities using tabulated sensitivity factors.

To measure the specific surface areas of NP Au foams, we used the Brunauer-Emmett-Teller method. Measurements were performed in a Fisons Sorptomatic 1900 apparatus on material degassed at 300 K and exposed to N_2_ at about 77 K.

Full details on experimental methods are given in Supporting Information SI. 1.

## Electronic supplementary material

Supplementary Information

## Data Availability

Data supporting the findings discussed in the study are available from the authors on request.
