# Supplementary material for: Ag surface segregation in nanoporous Au catalysts during CO oxidation
Source: Sci Rep. 2018 Oct 12;8:15208. doi: 10.1038/s41598-018-33631-4 (PMC6185915; doi:10.1038/s41598-018-33631-4)
Supplement: Supplementary file 1 — Supplementary Information [file 41598_2018_33631_MOESM1_ESM.pdf]

Ag surface segregation in nanoporous Au catalysts during CO oxidation

Giorgio Pia[1,*], Elisa Sogne[2], Andrea Falqui[2] and Francesco Delogu[1]

Dipartimento di Ingegneria Meccanica, Chimica, e dei Materiali, Università degli Studi di Cagliari, via Marengo 2, 09123 Cagliari, Italy.

King Abdullah University of Science and Technology (KAUST), Biological and Environmental Sciences and Engineering (BESE) Division, NABLA Lab, 23955-6900 Thuwal, Saudi Arabia

[*] Corresponding author: Giorgio Pia – giorgio.pia@dimcm.unica.it

Supporting Information

SI. 1. Experimental methods

NP Au foams were fabricated starting from a parent $Ag_{70}Au_{30}$ alloy. The alloy was synthesized using Ag and Au commercial powders with particle size below 45 μm and 99.99% purity, purchased from Sigma Aldrich. Elemental powders were mixed to obtain homogeneous powder mixtures with the desired $Ag_{70}Au_{30}$ composition. In each experiment, 8 g of powder mixture were sealed inside a hardened steel cylindrical reactor together with a single stainless steel ball of 12 g. Powder was invariably handled under inert Ar atmosphere with oxygen and humidity contents below 2 ppm. The reactor was fixed on the mechanical arm of a SPEX Mixer/Mill 8000, which was then operated at about 875 rpm. Milling time was suitably selected to allow the complete mutual dissolution of Ag and Au, and the formation of a homogeneous, chemically disordered crystalline solid solution. Finally, processed powders were annealed at 523 K for 10 h under Ar flux conditions in a tubular quartz reactor placed inside a laboratory Nabertherm N60/ER furnace.

Structural transformations induced by mechanical alloying and subsequent annealing were monitored by X-ray diffraction (XRD). XRD patterns were collected from disk-shaped specimens of compacted powders with a Miniflex II Rigaku diffractometer, equipped with Cu K_α radiation, over the scattering angle 2θ range between 20° and 90°, using discrete angular displacements of 0.01° and acquisition times of 1 min per point. The Rietveld method was used to analyze quantitatively the XRD patterns, and gain information on the alloy microstructure and average chemical composition [1].

XRD patterns indicate that mechanical alloying takes place gradually. A sigmoidal kinetic curve describes the mutual dissolution of the two elements, which finally results in the formation of a nanostructured face-centered cubic phase with unit cell parameter of about 0.4083 nm with no residual Ag or Au crystalline phases. Rietveld analysis suggests a grain size around 30 nm.

Annealing at 523 K makes the grains grow up to the micrometer scale, leaving the unit cell parameter unaffected. The 0.4083 nm value coincides with the one predicted by the Vegard's law [2] combining the unit cell parameters of pure Ag and Au equal to about 0.4085 and 0.4078 nm respectively [3].

Annealed $Ag_{70}Au_{30}$ powders were compacted into cylindrical pellets about 1 mm thick and 1 cm in diameter using a load of 10 MPa. The pellets were immersed in a 100 ml aqueous solution containing nitric acid at 70%. Temperature was kept constant at 300 K. Free chemical etching induced the selective dissolution of Ag, with a consequent increase of Ag^+ ion concentration in solution and a decrease of residual Ag content in the solid. XRD was used to follow the latter process, whereas the former was followed by argentometric titrations of aqueous solutions according to the Volhard method [4]. Results support each other, indicating the monotonic decrease of the Ag content in NP Au foams as discussed in previous work [5]. Etching was interrupted after 6.5 h to leave a residual Ag content of about 3.8 at.%. Once extracted from the acid solution, the etched pellets were immersed in distilled water. Water rinsing was performed to remove residual acid from pores.

A laboratory precision balance able to read four decimal places to the right of the decimal point was used to weigh dry etched pellets. The observed mass loss indicates the almost complete removal of Ag from the solid. Simple calculations based on residual mass and pellet geometry allow estimating the relative density of fabricated NP Au foams. In agreement with previous work [5], it approximately equals 0.294.

The structure of NP Au foams was investigated by scanning and transmission electron microscopy (SEM and TEM). TEM observation was performed on samples placed on Cu grids using a FEI Tecnai G12 TEM microscope. TEM was mainly used to support SEM observation for NP Au foams with ligament thickness smaller than 20 nm. Coarser structures were studied mostly using a Zeiss EVO LS15 SEM apparatus. NP Au characteristic lengths were estimated averaging over 50 different measurements, therefore involving measurements for 50 different ligaments and nodes. In turn, this required using a minimum of 5 and a maximum of 10 SEM and/or TEM micrographs per NP Au foam, depending on its structural features.

Catalytic runs were performed inside a tubular quartz reactor 1 cm in diameter under continuous flow conditions. NP Au foams were immobilized approximately at half reactor length using two porous inert alumina disks, and heated up to 353 K for 3 min to remove absorbed gases. Then, temperature was reduced to the desired value and kept constant while the reactant gaseous mixture of 1% CO, 10% O_2, and 89% N_2 was injected into the reactor at a space velocity of 120 dm^3 h^{-1} per gram of catalyst at 1 atm pressure. Catalytic tests were performed at 273, 283, 293 and 303 K. Relative amounts of CO and CO_2 were measured using a Shimadzu GC-8A gas chromatograph directly connected with the tubular quartz reactor.

Control experiments were performed exposing NP Au foams to a gaseous mixture of 10% O_2 and 90% N_2 and to pure N_2 under the same above-mentioned experimental conditions. These experiments aimed at evaluating the role of gaseous atmosphere in Ag surface segregation processes. In particular, a

comparison between the results of experiments performed in the presence and in the absence of CO allows highlighting the effects of catalytic transformations on overall NP Au behavior, whereas exposure to gaseous O_2 and pure N_2 allows isolating chemical and compositional effects related to O_2.

Specific surface area of NP Au foams was measured using the Brunauer-Emmett-Teller method. Measurements were performed on pristine NP Au foams and after selected time intervals of exposure to gaseous atmosphere. Physisorption experiments were carried out using a Fisons Sorptomatic 1900 apparatus. NP Au foams were degassed at 300 K and exposed to N_2 at about 77 K.

X-ray photoelectron spectroscopy (XPS) was used to estimate relative chemical composition of NP Au surfaces and neighboring regions. Measurements were performed at room temperature under ultra-high vacuum conditions using a Perkin Elmer Phi 5600 ESCA spectrometer equipped with a Mg K_α X-ray source at a take-off angle of 45°. Power dissipated over the exposed NP Au sample area was around 164 W. Following literature [6], investigation was focused on Ag(3d) and Au(4f) binding energy regions. The characteristic C(1s) peak at 284.6 eV was used to calibrate binding energies of Ag(3d) and Au(4f) electronic states. Relative surface composition was estimated from measured intensities of Ag(3d) and Au(4f) peaks using tabulated sensitivity factors. In particular, relative Ag surface concentration was calculated as the ratio between the number of surface Ag atoms and the total number of surface atoms. It should be noted that this procedure assumes a constant composition of the top few layers. Measurements were suitably repeated to estimate the error affecting relative Ag and Au surface concentration, which was found to be around 5%.

SI. 2. XPS measurements

XPS was used to detect changes in surface compositional profiles of Ag and Au species possibly induced by the exposure of NP Au foams to a certain gaseous atmosphere. A sequence of XPS patterns concerning the NP Au catalyst working at 283 K is shown in Fig. SI. 2. 1. Pristine NP Au foams exhibit a Au(4f) spectrum with main peaks at 84.5 and 88.2 eV that can be ascribed, respectively, to Au($4f_{7/2}$) and Au($4f_{5/2}$) contributions from metallic Au, and shoulders at 86.2 and 89.9 eV related to the presence of Au^{3+} species due to the formation of Au_2O_3 oxide [6,7]. The Ag(3d) spectrum also exhibits main peaks and shoulders. Main peaks at 368.6 and 374.8 eV identify Ag($3d_{5/2}$) and Ag($3d_{3/2}$) signals from metallic Ag, whereas the shoulders at 367.2 and 373.4 eV originate from Ag^{+} species consequent to the formation of Ag_2O [6].

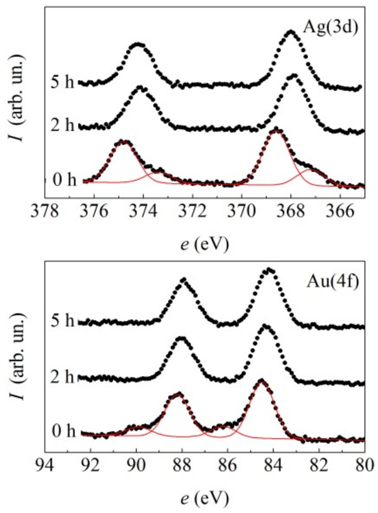

Fig. SI. 2. 1. The Ag(3d) and Au(4f) binding energy regions of the XPS patterns of NP Au foams exposed to reactive gases for 0, 2 and 5 h at 283 K. Intensity, *I*, is plotted as a function of binding energy, *e*. Deconvoluted spectra components for pristine NP Au foams are shown.

Exposure of NP Au foams to reactive gases determines the rapid disappearance of shoulders in both Au(4f) and Ag(3d) binding energy regions, and the simultaneous shift of main peaks towards lower binding energies. In principle, the observed down-shift of Au(4f) peaks can be related to two different contributions, namely the coarsening of NP Au ligaments and the reduction of oxidized Au species [6,8,9]. However, pristine NP Au foams have ligaments about 15 nm in diameter, coarse enough to make size effects negligible. Thus, reduction of positively charged Au species consequent to chemical transformations at the NP Au surface seems the most reasonable alternative [6]. A similar explanation can be invoked also for Ag(3d) peaks. Indeed, the final binding energy of Ag(3d) peaks corresponds to the normal value exhibited by metallic Ag [6,10,11].

The above-mentioned considerations hold true for all NP Au catalysts working at different temperatures. XPS signals emitted by NP Au foams exposed to reactive gases at different temperatures are strictly comparable. Peaks keep the same shape, with differences in intensity simply related to the different Ag surface segregation rates.

SI. 3. Control experiments

Experimental evidence concerning NP Au foams exposed to 1% CO, 10% O_2 and 89% N_2 gaseous mixture suggests that microscopic processes different from thermal diffusion mediate the first stage of Ag surface segregation. Other factors are at work, somehow facilitating Ag migration from bulk to surface.

In this regard, favorable chemical interactions between Ag and adsorbed O_2 can be expected to play a role. Indeed, Ag has been already shown to segregate at the surface of different alloys in the presence of gaseous O_2 [12-17]. To verify this hypothesis, pristine NP Au foams were exposed to a gaseous mixture of 10% O_2 and 90% N_2 under the same flow and temperature conditions utilized in the presence of CO. Being CO absent, no catalytic transformation can occur, but only interactions between the surface of NP Au foams and gaseous species.

The relative surface concentration of Ag atoms, χ, is shown in Fig. SI. 3. 1a as a function of time, *t*. In all investigated cases, χ grows monotonically and the growth rate increases with temperature. Therefore, Ag surface segregation processes occur as well in the absence of CO, induced by interactions between O_2 and Ag at the surface of NP Au foams.

Kinetic curves have shape similar to those observed in the presence of CO, and shown in Fig. 3a in the main text. However, time scales are considerably different. In the presence of CO, Ag surface segregation is almost complete after about 5 h, whereas time intervals approximately 10 times longer are involved in the absence of CO. This time scale is compatible with the slow χ increase observed in the second stage of Ag surface segregation processes taking place in the presence of CO, but not with the rapid increase during the first stage.

The kinetics of Ag surface segregation processes in the absence of CO is satisfactorily described by the equation below

$$\chi = \chi_{in} + (\chi_{fin} - \chi_{in})[1 - \exp(-k\,t)], \qquad \text{(SI. 3. 1)}$$

where χ_{in} is the initial relative Ag surface concentration, χ_{fin} the final relative Ag surface concentration, and k the apparent rate constant. As shown in Fig. SI. 3. 1b, the plot of $\ln[1-(\chi - \chi_{in})/(\chi_{fin} - \chi_{in})]$ as a function of time, *t*, is invariably linear. The capability of Eq. SI. 3. 1 of interpolating satisfactorily the entire data set indicates that the kinetics of Ag surface segregation in the absence of CO is single-stage. Accordingly, whereas χ_{in} can be set equal to 0.073, best χ_{fin} values range between 0.26 and 0.31, which correspond to the χ values attainable at the end of the Ag surface segregation process.

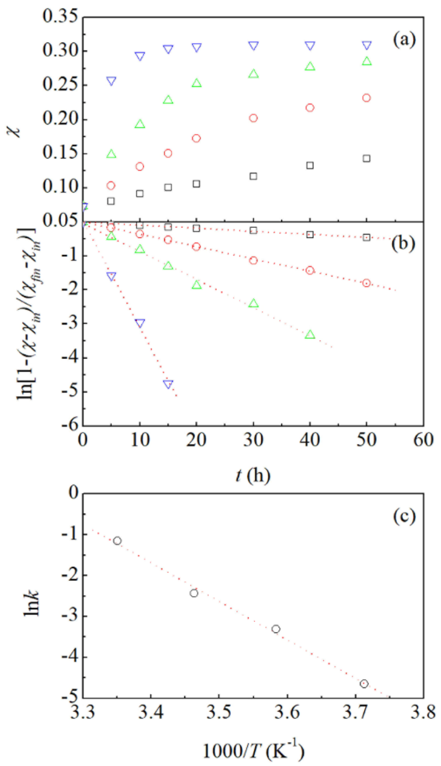

Fig. SI. 3. 1. The relative surface concentration of Ag atoms, χ, as a function of time, *t*. Data refer to NP Au foams exposed to a gaseous mixture of 10% O_2 and 90% N_2 at 273 (□), 283 (○), 293 (△) and 303 (▽) K.

Best fitting the linear plots in Fig. SI. 3. 1b allows estimating the apparent rate constants, *k*, of the Ag surface segregation process in the absence of CO. The linearity of semi-logarithmic plots shown in Fig. SI. 3. 1c reveal the Arrhenius-like behavior of rate constants, which exhibit an apparent activation energy, E_{a,O_2}, approximately equal to 78.3 kJ mol^{-1}. This value is almost perfectly half way between the value of about 104.5 kJ mol^{-1} for bulk diffusion of Ag atoms in Au [18] and the one of about 62 kJ mol^{-1} for Au atom surface diffusion [19]. Therefore, it is compatible with the diffusion of Ag atoms in bulk Au.

In the absence of CO, Ag surface segregation is not accompanied by coarsening. SEM micrographs in Fig. SI. 3. 2 show that the structure of NP Au foams keeps substantially unaltered. Indeed, ligament diameter are around 20 nm after 50 h of exposure to the 10% O_2 and 90% N_2 gas mixture.

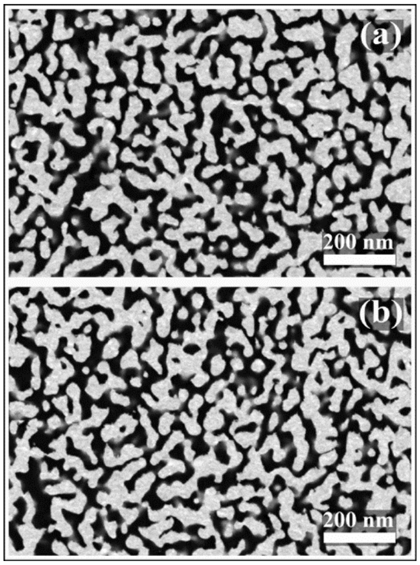

Fig. SI. 3. 2. SEM micrographs of NP Au foams exposed to the 10% O_2 and 90% N_2 gas mixture for (a) 20 and (c) 50 h at 303 K.

Additional experiments were performed to evaluate the NP Au behavior in the absence of both CO and O_2. To this aim, NP Au foams were exposed to pure N_2 under the same flow and temperature conditions used for other experiments.

The relative surface concentration of Ag atoms, χ, is shown in Fig. SI. 3. 3 as a function of time, *t*. Irrespective of temperature, χ keeps constant at the initial value. Therefore, Ag surface segregation does not take place in the presence of gaseous N_2 only.

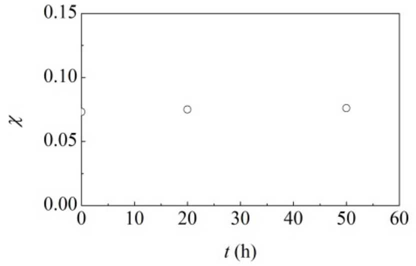

Fig. SI. 3. 3. The relative surface concentration of Ag atoms, χ, as a function of time, *t*. Data refer to NP Au foams exposed to pure N_2 at 303 K.

Analogously, SEM micrographs shown in Fig. SI. 3. 4 clearly indicate that the structure of NP Au foams remains unaffected.

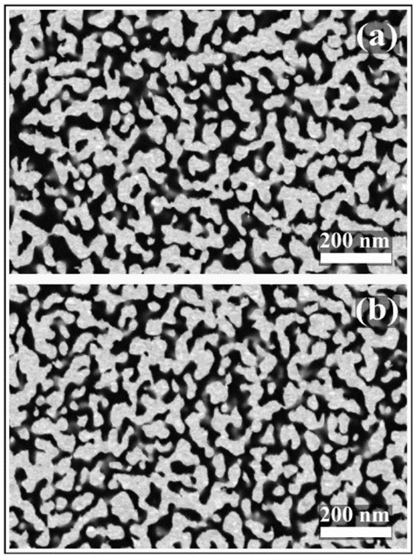

Fig. SI. 3. 4. SEM micrographs of NP Au foams exposed to pure N_2 for (a) 20 and (c) 50 h at 303 K.

SI. 4. Volume affected by coarsening

Experimental evidence summarized in Fig. 4a in the main text demonstrates that specific surface area, S_{sp}, average ligament thickness, *s*, and density of bulk Au, ρ_{sol}, satisfy the relationship $S_{sp}\, s\, \rho_{sol} = C$. The *C* value of about 3.4 suggests for NP Au foams coarsening at different temperature the same gyroidal character. The invariance of this morphological feature in spite of the general increase of characteristic lengths allows a rough evaluation of the volume involved in the mass transport processes mediating coarsening.

To this aim, it is useful to refer to the total surface area, *S*, of NP Au foams. By definition, $S = S_{sp}\, m_{tot}$, where m_{tot} is the total mass of Au. Since catalytic transformations and coarsening processes do not affect the total mass of NP Au catalysts, m_{tot} is a constant quantity.

The total surface area can be regarded as the sum of the properly defined surface areas of individual ligaments and nodes constituting NP Au foams. Since coarsening repeatedly involves the progressive thinning of ligaments and their final pinch-off, with consequent pore growth, the resulting reduction of total surface area can be ideally related to the variation in the total number of ligaments and nodes. A convenient choice to account for such morphological modification is to consider the mass affected, m^*. This allows suitably tackling the difficulty of evaluating the number of ligaments and nodes actually involved in the reduction of total surface area from the initial S_{in} value to the *S* one reached after a time *t* based on their particular shapes and characteristic lengths. The two surface areas necessarily satisfy the relationships $S_{in}\, s_{in}\, \rho_{sol} / m_{tot} = C$ and $S\, s\, \rho_{sol} / m_{tot} = C$, where s_{in} and *s* are, respectively, the ligament thickness in pristine NP Au foams and after any given time *t*.

According to above-mentioned considerations, surface area reduction from S_{in} to *S* can be explained by the disappearance of a certain number of structural elements and the redistribution of the

corresponding mass m^* over the surface area left unaffected by the local coarsening episode, as schematically shown in Fig. SI. 4. 1.

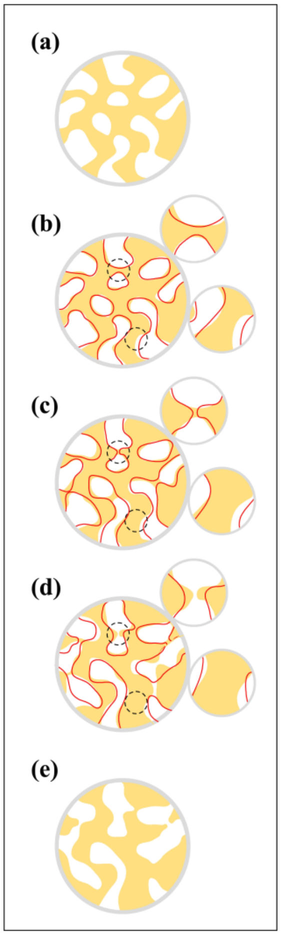

Fig. SI. 4. 1. Schematic description of coarsening. The structure of pristine NP Au, shown in (a), gradually evolves into structures in (b), (c), (d) and (e). In each picture, the red line shows the NP Au structure compared with the one at the previous stage. The thinning of some ligaments and the simultaneous coarsening of others mediate morphological evolution. The small circular insets show two representative cases. Starting from (b), the upper inset shows the thinning of a ligament and the final pinch-off event, which occurs in (d). The lower inset shows, instead, the coarsening of a ligament.

If S_{in} and S are sufficiently close, redistributed mass m^* is definitely smaller than m_{tot}. Under such circumstances, the difference between S_{in} and S can be entirely ascribed to the surface area disappeared consequent to the mass m^* redistribution. To a first approximation, this latter quantity can be equaled to the product between m^* and the initial specific surface area, equal to $S_{in}\, s_{in}\, \rho_{sol} / m_{tot}$. It follows that $S = S_{in} - S_{in}\, s_{in}\, \rho_{sol}\, m^* / m_{tot}$. Replacing S_{in} and S with their expressions as a function of ligament size, the latter equation can be rearranged into

$$\frac{C\, m_{tot}}{s\, \rho_{sol}} = \frac{C\, m_{tot}}{s_{in}\, \rho_{sol}} - \frac{C}{s_{in}\, \rho_{sol}} m^* . \qquad \text{(SI. 4. 1)}$$

In turn, Eq. SI. 4. 1 simplifies to

$$m^* = m_{tot}\left(1 - \frac{s_{in}}{s}\right). \qquad \text{(SI. 4. 2)}$$

Therefore, the volume affected by coarsening processes causing ligaments to increase their average thickness from s_{in} to s is approximately equal to

$$v_c \approx \frac{m_{tot}}{\rho_{sol}}\left(1 - \frac{s_{in}}{s}\right). \qquad \text{(SI. 4. 3)}$$

SI. References

1. Lutterotti, L., Ceccato, R., Dal Maschio, R. & Pagani, E. Quantitative Analysis of Silicate glass in Ceramic Materials by the Rietveld Method. *Mater. Sci. Forum* **278–281,** 87–92 (1998).

2. Denton, A. R. & Ashcroft, N. W. Vegard's law. *Phys. Rev. A* **43,** 3161–3164 (1991).

3. *Smithells Metals Reference Book*. (eds. Brandes, E. A. Brook, G. B. Butterworth-Heinemann, 1992).

4. Harris, D. C. *Quantitative Chemical Analysis*. (W. H. Freeman Publishing, 2003).

5. Pia, G., Mascia, M. & Delogu, F. Kinetics of nanoporous Au formation by chemical dealloying. *Scr. Mater.* **76,** 57–60 (2014).

6. Wang, L.-C. *et al.* Catalytic activity of nanostructured Au: Scale effects versus bimetallic/bifunctional effects in low-temperature CO oxidation on nanoporous Au. *Beilstein J. Nanotechnol.* **4,** 111–128 (2013).

7. Klyushin, A. Y., Rocha, T. C. R., Havecker, M., Knop-Gericke, A. & Schlogl, R. A near ambient pressure XPS study of Au oxidation. *Phys. Chem. Chem. Phys* **16,** 7881–7886 (2014).

8. Fu, Q., Saltsburg, H. & Flytzani-Stephanopoulos, M. Active Nonmetallic Au and Pt Species on Ceria-Based Water-Gas Shift Catalysts. *Scien.* **301,** 935–938 (2003).

9. Deng, W., Carpenter, C., Yi, N. & Flytzani-Stephanopoulos, M. Comparison of the activity of Au/CeO2 and Au/Fe2 O3 catalysts for the CO oxidation and the water-gas shift reactions. *Top. Catal.* **44,** 199–208 (2007).

10. Hoflund, G. B. & Hazos, Z. F. Surface characterization study of Ag, AgO , and Ag2 O using x-ray photoelectron spectroscopy and electron energy-loss spectroscopy. *Phys. Rev.B* **62,** 126–133

(2000).

11. Wang, A., Liu, J., Lin, S. D., Lin, T. & Mou, C. A novel efficient Au – Ag alloy catalyst system: preparation, activity, and characterization. *J. Catal.* **233,** 186–197 (2005).

12. Kitchin, J. R., Reuter, K. & Scheffler, M. Alloy surface segregation in reactive environments: A first-principles atomistic thermodynamics study of Ag3Pd(111) in oxygen atmospheres. *Phys. Rev.B* **77,** 075437 (2008).

13. Zafeiratos, S., Piccinin, S. & Teschner, D. Alloys in Catalysis: Phase Separation and Surface Segregation Phenomena in Response to the Reactive Environment. *Catal. Sci. Technol* **2,** 1787–1801 (2012).

14. Svenum, I., Herron, J. A., Mavrikakis, M. & Venvik, H. J. Adsorbate-induced segregation in a PdAg membrane model system: Pd3Ag(1 1 1). *Catal. Today* **193,** 111–119 (2012).

15. Guesmi, H. Theoretical insights on the effect of reactive gas on the chemical ordering of gold-based alloys. *Gold bull* **46,** 213–219 (2013).

16. Déronzier, T., Morfin, F., Lomello, M. & Rousset, J. Catalysis on nanoporous gold – silver systems: Synergistic effects toward oxidation reactions and influence of the surface composition. *J. Catal.* **311,** 221–229 (2014).

17. Montemore, M. M., Madix, R. J. & Kaxiras, E. How Does Nanoporous Gold Dissociate Molecular Oxygen? *J. Phys. Chem. C* **120,** 16636–16640 (2016).

18. Mallard, W. C., Gardner, A. B., Bass, R. . & Slifkin, L. M. Self-Diffusion in Silver-Gold Solid Solutions. *Phys. Rev.* **129,** 617–625 (1962).

19. Chen-Wiegart, Y. K. *et al.* Structural evolution of nanoporous gold during thermal coarsening. *Acta Mater.* **60,** 4972–4981 (2012).
